# Abnormalities of the Ventilatory Equivalent for Carbon Dioxide in Patients with Chronic Heart Failure

**DOI:** 10.1155/2012/589164

**Published:** 2012-04-29

**Authors:** Lee Ingle, Rebecca Sloan, Sean Carroll, Kevin Goode, John G. Cleland, Andrew L. Clark

**Affiliations:** ^1^Department of Sport, Health & Exercise Science, University of Hull, Cottingham Road, Kingston-upon-Hull HU6 7RX, UK; ^2^Department of Cardiology, Hull York Medical School, Daisy Building, University of Hull, Castle Hill Hospital, Cottingham, Kingston-upon-Hull HU16 5JQ, UK

## Abstract

*Introduction*. The relation between minute ventilation (VE) and carbon dioxide production (VCO_2_) can be characterised by the instantaneous ratio of ventilation to carbon dioxide production, the ventilatory equivalent for CO_2_ (VEqCO_2_). We hypothesised that the time taken to achieve the lowest VEqCO_2_ (time to VEqCO2 nadir) may be a prognostic marker in patients with chronic heart failure (CHF). *Methods*. Patients and healthy controls underwent a symptom-limited, cardiopulmonary exercise test (CPET) on a treadmill to volitional exhaustion. *Results*. 423 patients with CHF (mean age 63 ± 12 years; 80% males) and 78 healthy controls (62% males; age 61 ± 11 years) were recruited. Time to VEqCO2 nadir was shorter in patients than controls (327 ± 204 s versus 514 ± 187 s; *P* = 0.0001). Univariable predictors of all-cause mortality included peak oxygen uptake (*X* ^2^ = 53.0), VEqCO_2_ nadir (*X* ^2^ = 47.9), and time to VEqCO_2_ nadir (*X* ^2^ = 24.0). In an adjusted Cox multivariable proportional hazards model, peak oxygen uptake (*X* ^2^ = 16.7) and VEqCO_2_ nadir (*X* ^2^ = 17.9) were the most significant independent predictors of all-cause mortality. *Conclusion*. The time to VEqCO_2_ nadir was shorter in patients with CHF than in normal subjects and was a predictor of subsequent mortality.

## 1. Introduction

Cardiopulmonary exercise testing (CPET) is used to stratify risk in patients with cardiorespiratory disease [1]. In patients with chronic heart failure (CHF), the normal linear relation between ventilation (VE) and carbon dioxide production (VCO_2_) is maintained, but the slope of the relation is greater than normal, so that, for a given volume of carbon dioxide production, the ventilatory response is greater [2–6]. Another way of characterising the relation between minute ventilation and carbon dioxide production is the instantaneous ratio of ventilation to carbon dioxide production, the ventilatory equivalent for CO_2_ (VEqCO_2_). Recently, we have shown that the lowest VEqCO_2_ (VEqCO_2_ nadir) provides greater prognostic value than other CPET-derived variables in patients with suspected CHF [7]. Other studies have reported that the lowest VEqCO_2_ has similar prognostic power to the VE/VCO_2_ slope derived from the whole of exercise [8]. 

During an incremental CPET, as exercise intensity increases, both VCO_2_ and VE increase linearly. However, VEqCO_2_ falls at the onset of exercise, possibly due to a reduction in dead space ventilation. Beyond the ventilatory compensation point (VCP), lactic acid production causes an increase in ventilation relative to carbon dioxide production, and thus the VEqCO_2_ rises. Although patients with CHF have the same pattern of VEqCO_2_ during exercise as normal subjects, with more severe heart failure, the increase in VEqCO_2_ towards the end of exercise becomes more marked [9]. In the most severely affected patients, VEqCO_2_ increases from the start of exercise [9]. We hypothesised that the time taken to reach VEqCO_2_ nadir would be shorter in patients with CHF compared to healthy controls and thus may be an important prognostic indicator.

## 2. Methods

The Hull and East Riding Ethics Committee approved the study, and all patients provided informed consent. We recruited consecutive patients referred to a community heart failure clinic with symptoms of breathlessness (NYHA functional class II-III) who were found to have left ventricular systolic dysfunction on investigation. Clinical information obtained included past medical history and drug and smoking history. Clinical examination included assessment of body mass index (BMI), heart rate, rhythm, and blood pressure. Patients were excluded if they were unable to exercise because of noncardiac limitations (such as osteoarthritis) or had significant respiratory disease (defined as a predicted FEV_1_ < 70%).

Heart failure was defined as the presence of current symptoms of HF, or a history of symptoms controlled by ongoing therapy, and impaired left ventricular systolic function. Left ventricular function was determined from 2D echocardiography which was carried out by one of three trained operators. Left ventricular function was assessed by estimation on a scale of normal, mild, mild-to-moderate, moderate, moderate-to-severe, and severe impairment and was assessed by a second operator blind to the assessment of the first; where there was disagreement on the severity of left ventricular (LV) dysfunction, the echocardiogram was reviewed jointly with the third operator and a consensus reached. Where possible, left ventricular ejection fraction (LVEF) was calculated using the Simpson's formula from measurements of end-diastolic and end-systolic volumes on apical 2D views, following the guidelines of Schiller and colleagues [10], and LVSD was diagnosed if LVEF was ≤45%. When LVEF could not be calculated, LVSD was diagnosed if LVEF ≤45 or there was at least “mild-to-moderate” impairment.

Patients underwent a symptom-limited, maximal CPET on a treadmill using the Bruce protocol modified by the addition of a Stage 0 (2.74 km*·*h^−1^ and 0% gradient) at the onset of exercise. Metabolic gas exchange was measured with an Oxycon Delta metabolic cart (VIASYS Healthcare Inc., Philadelphia, PA, USA). Peak oxygen uptake (pVO_2_) was calculated as the average VO_2_ for the final 30 s of exercise. The ventilatory anaerobic threshold (AT) was calculated by the V-slope method [11]. The gradient of the relationship between VE and VCO_2_ (VE/VCO_2_ slope) was calculated by linear regression analysis using data acquired from the whole test. The VEqCO_2_ relation was plotted from start to the finish of exercise. Each consecutive 30-second reading was averaged, and the lowest point was defined as the VEqCO_2_ nadir [7]. The time taken to reach the VEqCO_2_ nadir was reported in seconds (s). The peak respiratory exchange ratio (pRER) was calculated as the mean VCO_2_/VO_2_ ratio for the final 30 s of exercise. For comparative purposes, we also included a healthy control group who had no evidence of cardiac, respiratory, or musculoskeletal limitation. Healthy controls were randomly invited to participate from two local GP practices.

### 2.1. Statistical Analysis

We used SPSS (version 17.0) for statistical analysis. Continuous variables are presented as mean ± SD, and categorical data are presented as percentages. Continuous variables were assessed for normality by the Kolmogorov-Smirnov test. An arbitrary level of 5% statistical significance was used throughout (two tailed). An independent *t*-test was used to measure differences between CHF patients and healthy controls. All survivors were followed for a minimum of 12 months, and we therefore give the probability of 12-month survival. Receiver operator characteristic (ROC) curves were used to identify the value of the strongest predictor variables of survival to 12 months. We reported the area under the curve (AUC) with 95% confidence intervals (CI), sensitivity, specificity, and optimal cut-points in our ROC analysis. To define the optimal cut-point, we used the point closest to the upper left corner of the ROC curve, often known as the (0, 1) criterion.

All baseline variables (Table 1) were entered as potential univariable predictors of mortality using Cox analysis, and we adjusted for age, sex, BMI, aetiology of heart failure, and severity of LV dysfunction (none, trivial, mild, mild-to-moderate, moderate, moderate-to-severe, severe). Model building was based on backward elimination (*P* value for entry was <0.05; *P* value for removal >0.1). A multivariable Cox proportional hazards model using the backward likelihood ratio method was used to identify independent predictors of all-cause mortality from all significant candidate predictor variables. The outcome measure was all-cause mortality. Kaplan-Meier survival curves were plotted for the strongest candidate predictors; data were dichotomised by optimal cut-points.

## 3. Results

423 patients with CHF (mean age  63 ± 12  years; 80% males; LVEF 36 ± 6%; peak VO_2_ 22.3 ± 8.1 mL·kg^−1^·min^−1^; VE/VCO_2_ slope 34 ± 8) were included in the study. Of these, 75% were taking ACE inhibitors, 77% beta blockers, and 67% loop diuretics. Seventy eight healthy subjects (62% males; age  61 ± 11  years) were recruited as a control group. The healthy controls had a higher peak oxygen uptake, lower VE/VCO_2_ slope, and lower VEqCO_2_ nadir (Table 1). Time to VEqCO_2_ nadir was shorter in patients than controls (327 ± 204 s versus 514 ± 187 s; *P* = 0.0001) but was similar as a percentage of the total exercise duration in both groups (55 ± 23% versus 60 ± 17%; *P* = 0.077). We performed a subgroup analysis in 62 NYHA class III patients and found that the time to VEqCO_2_ nadir was significantly lower (199 ± 59 s) compared to other less symptomatic patients (344 ± 202 s; *P* < 0.0001). We also performed a subgroup analysis by sex and found that the time to VEqCO_2_ nadir was very similar between males (327 ± 209 s) and females (328 ± 94 s; *P* > 0.05; *n* = 85).

**Figure 1:**
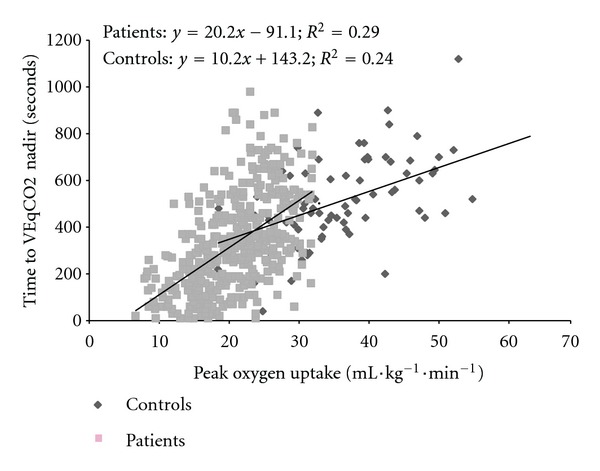
Relation between time to VEqCO2 nadir and peak oxygen uptake in patients with CHF and controls.

**Figure 2:**
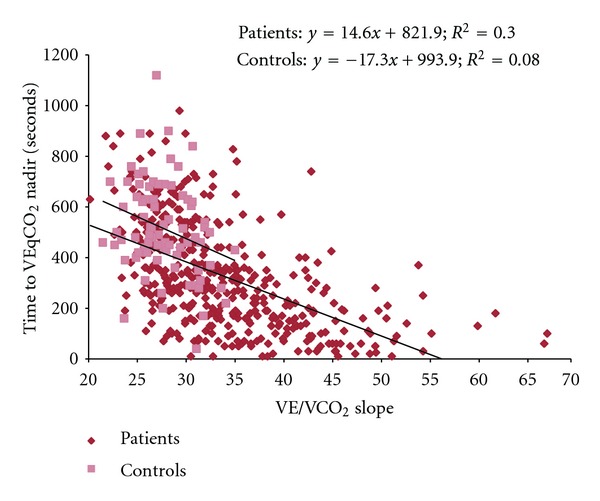
Relation between time to VEqCO_2_ nadir and VE/VCO_2_ slope in patients with CHF and controls.

In patients, time to VEqCO_2_ nadir correlated with age (*r* = −0.17; *P* = 0.0001) and LVEF (*r* = 0.24; *P* = 0.0001) but was not associated with BMI (*r* = 0.001; *P* = 0.98). Time to VEqCO_2_ nadir correlated with peak oxygen uptake (*r* = 0.59; *P* = 0.001) and showed an inverse association with both VE/VCO_2_ slope (*r* = −0.55; *P* = 0.001) and VEqCO_2_ nadir (*r* = −0.56; *P* = 0.001). Scatter plots showing the association between time to VEqCO_2_ nadir, peak oxygen uptake, and VE/VCO_2_ slope in patients and controls are shown in Figures 1 and 2.

One hundred and eighteen patients (28%) died during followup. The median followup in survivors was 8.6 ± 2.1 years. Univariable predictors of outcome derived from CPET are shown in Table 2. With the exception of resting heart rate, all candidate variables were significant univariable predictors. The strongest univariable predictors of all-cause mortality were peak oxygen uptake (*χ* ^2^ = 53.0), VEqCO_2_ nadir (*χ* ^2^ = 47.9), VE/VCO_2_ slope (*χ* ^2^ = 31.7), and time to VEqCO_2_ nadir (*χ* ^2^ = 24.0). In a Cox multivariable proportional hazards model adjusted for age, sex, BMI, and severity of LV dysfunction, peak oxygen uptake (*χ* ^2^ = 16.7; HR = 0.91; 95% CI 0.88–0.95; *P* = 0.0001) and VEqCO_2_ nadir (*χ* ^2^ = 17.9; HR = 1.12; 95% CI 1.04–1.20; *P* = 0.0001) were the most significant independent predictors of mortality.

**Figure 3:**
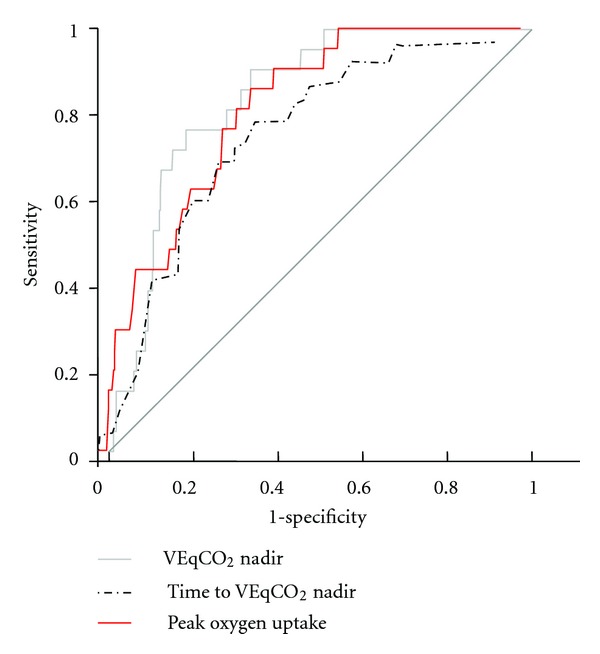
Receiver operating characteristic curve showing value of VEqCO_2_ nadir, time to VEqCO_2_ nadir, and peak oxygen uptake for predicting all-cause mortality at 12 months. VEqCO_2_ nadir: AUC = 0.81; *P* < 0.0001; 95% CI = 0.74–0.89; sensitivity = 86; specificity = 62; optimal cut-point = 33; time to VEqCO_2_ nadir: AUC = 0.75; *P* < 0.0001; 95% CI = 0.67–0.84; sensitivity = 81; specificity = 62; optimal cut-point = 250 s; peak VO_2_: AUC = 0.76; *P* < 0.0001; 95% CI = 0.67–0.85; sensitivity = 86; specificity = 57; optimal cut-point = 20 mL·kg^−1^·min^−1^.

**Figure 4:**
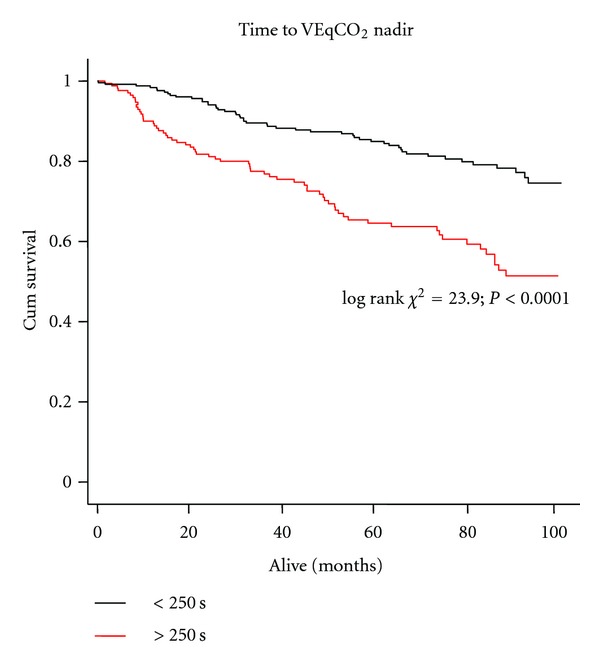
Kaplan-Meier survival curve showing time to VEqCO_2_ nadir-data dichotomised by optimal cut-points (<250 s; *n* = 170, event free survival 61%; ≥250 s *n* = 254 patients, event free survival 80%).

**Figure 5:**
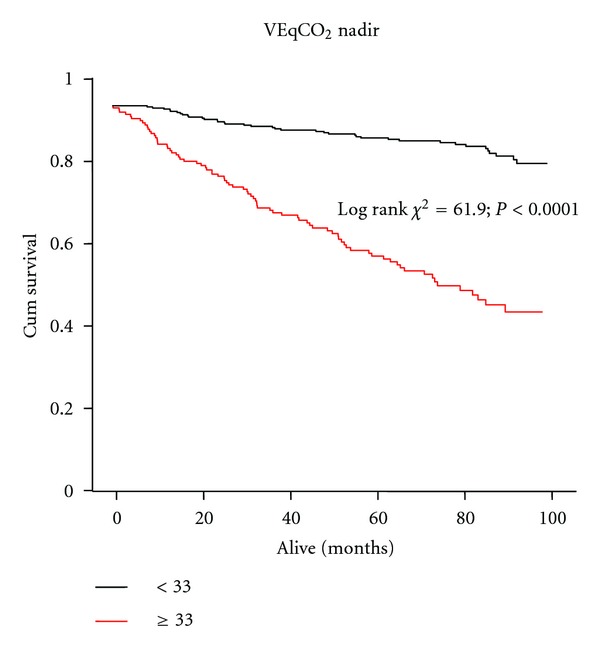
Kaplan-Meier survival curve showing VEqCO_2_ nadir-data dichotomised by optimal cut-points (<33 *n* = 252 patients, event free survival 85%; ≥33 *n* = 171 patients, event free survival 54%).

**Figure 6:**
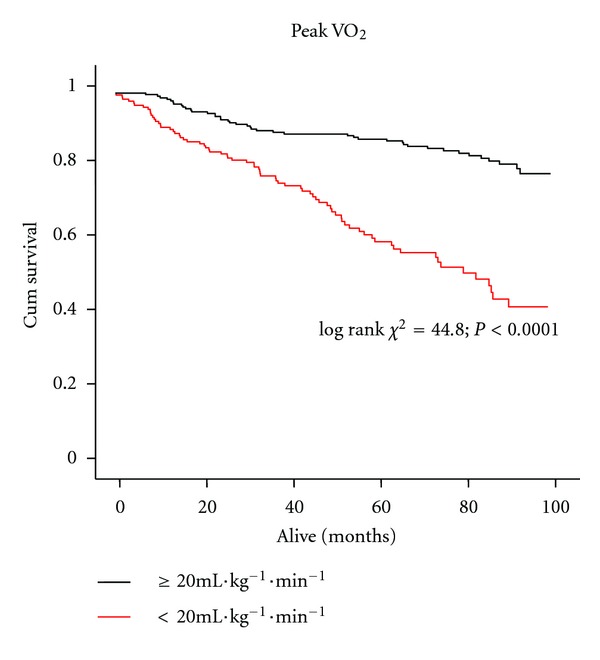
Kaplan-Meier survival curve showing peak VO_2_-data dichotomised by optimal cut-points (<20 mL·kg^−1^·min^−1^ *n* = 184 patients, event free survival 60%; ≥ 20 mL·kg^−1^·min^−1^ *n* = 239 patients, event free survival 82%).

ROC curve analysis of the relation between time to VEqCO_2_ nadir (and both VEqCO_2_ nadir and peak VO_2_) and all-cause mortality at 12 months is shown in Figure 3. Time to VEqCO_2_ nadir (AUC = 0.75; *P* < 0.0001; 95% CI = 0.67–0.84; sensitivity = 81; specificity = 62; optimal cut-point = 250 s); VEqCO_2_ nadir (AUC = 0.81; *P* < 0.0001; 95% CI = 0.74–0.89; sensitivity = 86; specificity = 62; optimal cut-point = 33); peak VO_2_ (AUC = 0.76; *P* < 0.0001; 95% CI = 0.67–0.85; sensitivity = 86; specificity = 57; optimal cut-point = 20 mL·kg^−1^·min^−1^) were similar in their relation to all-cause mortality at 12 months. Optimal cut-points determined from ROC analysis were used to construct Kaplan-Meier survival curves for time to VEqCO_2_ nadir (Figure 4), VEqCO_2_ nadir (Figure 5), and peak VO_2_ (Figure 6).

## 4. Discussion

We have shown that the time to VEqCO_2_ nadir is significantly lower in patients with CHF compared to controls. To our knowledge, no previous study has evaluated the prognostic value of time to VEqCO_2_ nadir. Sun and colleagues [12] showed that the lowest VEqCO_2_ (VEqCO_2_ nadir) was the most stable marker of ventilatory inefficiency in healthy controls. During maximal exercise testing, the VEqCO_2_ nadir was achieved at around the ventilatory anaerobic threshold and occurred during “moderate” exercise intensity. Both VE and VCO_2_ are linearly related up to the ventilatory compensation point (VCP). Beyond this point (during heavy to maximal exertion), an increase in VE relative to VCO_2_ is dependent upon the fall in pH and PaCO_2_ [12].

The exaggerated ventilatory response of patients with CHF is seen at the outset of exercise; that is, the VE/VCO_2_ slope is abnormal from the moment exercise starts. A wide variety of factors has been proposed as the reason for the increase in VE/VCO_2_ slope including an increased dead space and resultant “wasted” ventilation [13–15], early metabolic acidosis [16], and overactivation of chemoreceptors and ergoreceptors [17, 18]. The fall in the VEqCO_2_ at the onset of exercise is at least in part due to the reduction in fixed anatomical dead space ventilation as a proportion of total ventilation at the onset of exercise, but the increase after the plateau phase is due to a non-CO_2_ stimulus to ventilation, wheather lactate production or an alternative stimulus to ventilation, such as the ergoreflex [9, 19]. The shorter time to VEqCO_2_ nadir reflects the earlier onset (and more important influence of) the non-CO_2_ stimulus to ventilation in patients with CHF.

We found a strong relation between the time to VEqCO_2_ nadir and mortality. The time to VEqCO_2_ nadir was an important univariable predictor of all-cause mortality although it was outperformed by peak oxygen uptake and VEqCO_2_ nadir in a multivariable survival model. We have previously shown that peak oxygen uptake [20] and VEqCO_2_ nadir [7] are independent predictors of all-cause mortality in patients with CHF. Other investigators have also reported similar findings [8, 21].

A limitation of our study is that we do not have test-retest CPET data for individual patients/controls; therefore, we cannot determine the reproducibility of the time to VEqCO_2_ nadir in either healthy or diseased populations.

Cardiopulmonary exercise testing provides two broad types of prognostic variable: a measure of exercise capacity, such as peak VO_2_, reflecting the complex relation between pump, ventilator, and muscle extraction; and a measure of the ventilatory response to exercise, such as the VE/Vco_2_slope or time to VEqCO_2_ nadir, reflecting the abnormal stimulus to ventilation in CHF. The time to VEqCO_2_ nadir following maximal CPET was shorter in patients with CHF than in normal subjects and is a predictor of subsequent mortality.

## 5. Clinical Messages

- Cardiopulmonary exercise testing is becoming increasingly important for prescribing appropriate exercise training volumes in patients with cardiovascular disease including CHF.
- The ventilatory response to exercise is abnormal in patients with CHF compared to age-matched controls.
- Metabolic responses to exercise are important predictors of risk and should be monitored prior to and following a program of rehabilitation in patients with CHF.

## Figures and Tables

**Table 1:** Baseline clinical characteristics between CHF patients and healthy controls.

Variable (mean ± SD)	CHF patients	Healthy controls	*P* value*
*N*	423	78	—
Males (%)	80	62	0.0001*
Age (years)	63 (12)	61 (11)	0.529
BMI (kg·m^−2^)	28 (5)	26 (3)	0.001*
LVEF (%)	36 (6)	60 (6)	0.0001*
pVO_2_ (mL·kg^−1^·min^−1^)	22.3 (8.1)	36.2 (8.8)	0.0001*
VE/VCO_2_ slope (full)	33.8 (7.7)	27.7 (3.0)	0.0001*
VEqCO_2_ nadir	32.4 (6.2)	26.5 (3.0)	0.0001*
Time to VEqCO_2_ nadir(s)	327 (514)	514 (187)	0.0001*
AT (mL·kg^−1^·min^−1^)	15.2 (5.7)	23.4 (6.6)	0.0001*
Peak RER	1.07 (0.10)	1.08 (0.06)	0.223
Exercise duration (s)	564 (250)	881 (256)	0.0001*
Heart rate (rest) (beats*·*min^−1^)	76 (15)	72 (12)	0.079
Heart rate (peak) (beats*·*min^−1^)	136 (30)	165 (20)	0.0001*
Systolic BP (rest) (mmHg)	137 (25)	148 (20)	0.0001*
Systolic BP (peak) (mmHg)	172 (36)	199 (22)	0.0001*
Diastolic BP (rest) (mmHg)	84 (15)	90 (9)	0.0001*
Diastolic BP (peak) (mmHg)	93 (22)	101 (21)	0.003*
Loop diuretic (%)	67	—	—
ACE-I (%)	75	—	—
Beta-blocker (%)	77	—	—

BMI: body mass index; LVEF: left ventricular ejection fraction; pVO_2_: peak oxygen uptake; ACE-I: ACE inhibitor; peak RER: peak respiratory exchange ratio; AT: anaerobic threshold; BP: blood pressure; *differences between CHF and healthy controls, *P* < 0.05.

**Table 2:** Unadjusted univariable predictors of outcome (in order of Chi-square value).

Variables	*P* value	HR		95% CI	Chi-square
Peak oxygen uptake	0.0001	0.891	0.862	0.920	53.0
VEqCO_2_ nadir	0.0001	1.095	1.068	1.122	47.9
VE/VCO_2_ slope	0.0001	1.060	1.041	1.079	31.7
Time to VEqCO_2_ nadir*	0.0001	0.705	0.523	0.905	24.0
Heart rate at peak exercise	0.0001	0.995	0.978	0.992	18.5
Systolic blood pressure (rest)	0.001	0.986	0.978	0.994	12.0
Diastolic blood pressure (rest)	0.02	0.977	0.963	0.991	9.3
Heart rate (rest)	0.744	1.002	0.990	1.014	0.1

HR: hazard ratio; 95% CI: 95% confidence intervals; *adjusted HR associated with 10 s increase in time to VEqCO_2_ nadir.
